# Primary cutaneous apocrine gland carcinoma from areolar tissue in a male patient with gynecomastia: a case report

**DOI:** 10.1186/s13019-015-0319-5

**Published:** 2015-09-08

**Authors:** Kyung-Jin Seo, Jae-Jun Kim

**Affiliations:** 1 Departments of Hospital Pathology, Uijeongbu St. Mary’s Hospital, The Catholic University of Korea College of Medicine, Uijeongbu, South Korea; 2 Department of Thoracic and Cardiovascular Surgery, Uijeongbu St. Mary’s Hospital, The Catholic University of Korea College of Medicine, 271 Cheonbo Street, Uijeongbu, Gyeonggi-do 11765 South Korea

## Abstract

Primary cutaneous apocrine gland carcinoma, which is a type of sweat gland carcinoma, is an extremely rare type of cancer. Clinical courses of this type of cancer usually progress slowly but can, occasionally, be associated with rapid progression. This case report describes a 53-year-old Korean man with primary cutaneous apocrine gland carcinoma that arose from an apocrine gland in the areola tissue. The patient visited our hospital because of a large, painful chest wall mass beneath the right nipple. The mass had been present for more than eight years but had grown rapidly over the past few months. The patient was initially diagnosed with a benign cystic mass, and we performed a wide excision with a clear margin and without lymph node dissection. The mass was a well-encapsulated cystic lesion that contained old blood material, and there was no invasion into the surrounding tissue. The final pathology showed that the mass was a primary cutaneous apocrine gland carcinoma that arose from the areola apocrine sweat gland, not from the breast parenchymal tissue. Herein, we report an extremely rare chest wall mass unfamiliar to thoracic surgeons.

## Background

Primary cutaneous apocrine gland carcinoma (PCAGC) is an extremely rare malignant tumor of a skin appendage [1–4]. It most commonly develops in the axilla but can develop in any locations containing apocrine sweat glands [1–4]. Herein, we report a case of ingrowing primary cutaneous apocrine gland carcinoma that arose from an apocrine gland in the areola of a male patient with gynecomastia.

## Case report

**Fig. 1:**
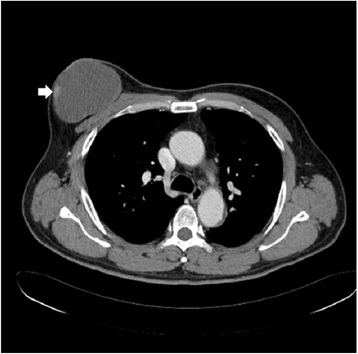
Chest CT shows a 9.3 × 6.7 cm, well-defined, thin-walled cystic mass in the right subareolar region with nodular enhancing lesions in the lateral wall, suggesting a benign soft tissue mass such as a large epidermal inclusion cyst

**Fig. 2:**
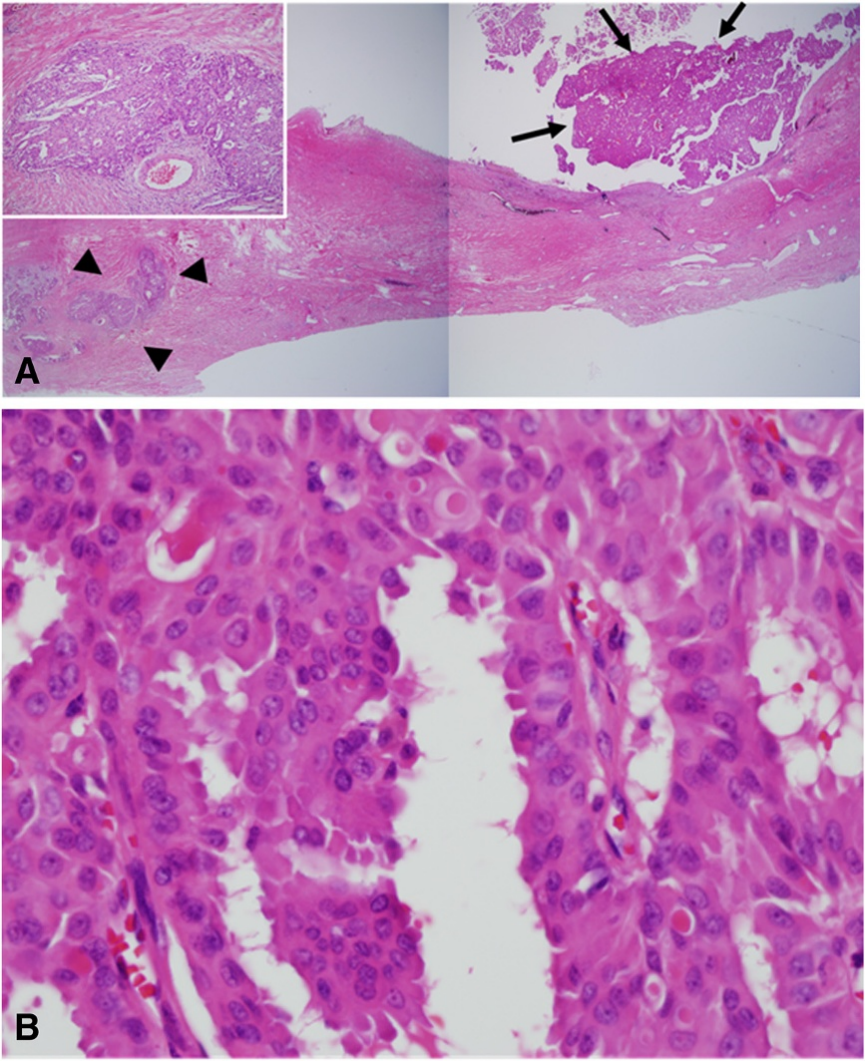
Microscopic findings show an intracystic papillary projection composed of some ill-defined tumor nests in the fibrotic wall, representing the in situ lesion of the tumor (black arrows, **a**, H&E, 10×). Note the invasive components (black arrowheads, **a**, H&E) adjacent to the in situ lesion (A 10×; inset, 100×). The tumor cells have abundant eosinophilic cytoplasm and vesicular nuclei with focal “apocrine-like” decapitation secretion (**b**, H&E, 400×), suggesting apocrine gland carcinoma

**Fig. 3:**
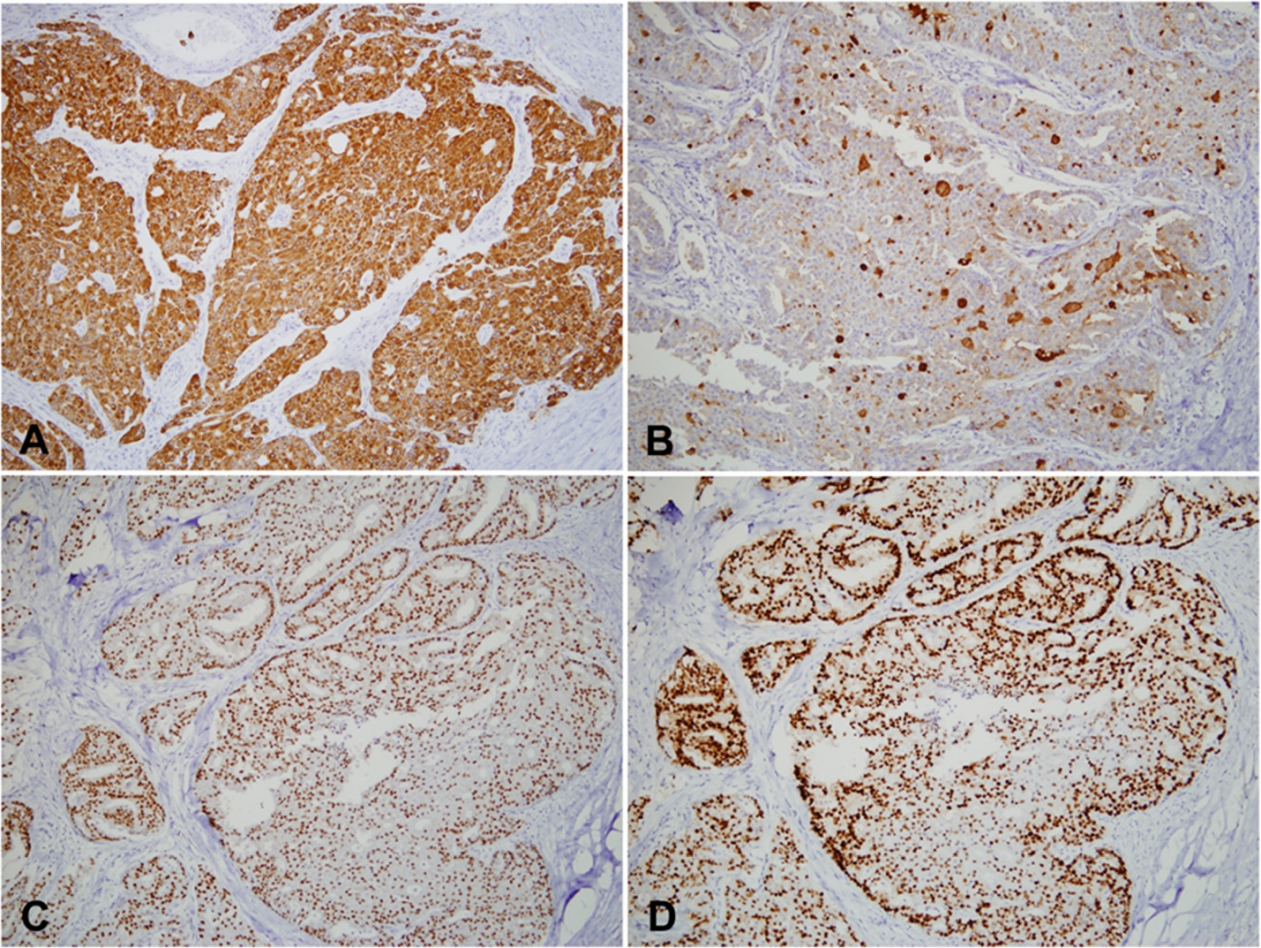
Immunohistochemistry findings show that the tumor cells are positive for cytokeratin AE1/AE3 (**a**) and gross cystic disease fluid protein (GCDFP)-15 (**b**). The tumor cells also express estrogen (**c**) and progesterone receptors (**d**) (a–d, 200×)

A 53-year-old Korean man presented to our hospital with a painful, large, round chest wall mass beneath the right nipple measuring about 10 × 10 cm in size. The skin overlying the mass was reddish and hyper-pigmented. The mass had been present for more than 8 years but had grown rapidly over the past few months. There were no palpable lymph nodes and no other breast masses on physical exams. A chest CT showed no remarkable lymph nodes and a 9.3 × 6.7 cm, well-defined, thin-walled, cystic mass with some nodular enhancing lesions in the lateral wall of the mass. This suggested a benign soft tissue mass, such as a large epidermal inclusion cyst (Fig. 1). Because we considered the mass to be a benign cystic mass, we excised it without lymph node dissection. We were able to save the nipple because the mass was limited to the subcutaneous layer beneath the areolar tissue. The mass was a well-encapsulated cystic lesion that contained old blood material, and there was no gross invasion into the surrounding tissue. Microscopic examination revealed an intracystic papillary projection (focal area with papillary projection), representing in situ carcinomatous lesions with apocrine features. A microscopic focus of the invasive carcinoma was identified in the cystic wall adjacent to the in situ lesions (Fig. 2a). Tumor cells had abundant eosinophilic cytoplasm and vesicular hyperchromatic nuclei with focal decapitation secretion (Fig. 2b). Most tumor cells were located within the in situ lesions and in the papillary projections, and the tumor tissue focally invaded the cystic wall adjacent to the papillary projections. However, the tumor had not invaded the surrounding tissue, such as subcutaneous tissue, muscle, or dermis (Fig. 2). Serial sections of the whole resected specimen failed to show breast parenchymal tissues. Immunohistochemical study showed that the tumor cells were positive for cytokeratin AE1/AE3, gross cystic disease fluid protein (GCDFP)-15 (Fig. 3a, b), and estrogen and progesterone receptors (Fig. 3c, d). Based on these findings, the preliminary pathological diagnosis was carcinoma with apocrine features. Clinical evaluations, including positron emission tomography-computed tomography (PET-CT), chest CT, abdominal and pelvic CT, colonoscopy, and duodenoscopy, were performed in order to exclude other potential primary malignancies, and no other potential primary malignancy was identified. Based on these characteristic features, we diagnosed the tumor as primary cutaneous apocrine gland carcinoma (cribriform and cystic form). The postoperative course was uneventful, and at present, 36 months after the surgery, no recurrence or metastasis has been identified, and the patient has not received any adjuvant therapy.

## Discussion

Primary cutaneous apocrine gland carcinoma (PCAGC), a unique type of malignant sweat gland tumor, is an extremely rare malignancy that originates from either normal or modified apocrine glands [1–4]. It usually develops in the axilla but can be found in all body sites containing apocrine sweat glands [1–4]. The clinical progression can vary, and PCAGC can range from slow-growing and relatively indolent to aggressive, rapidly progressive tumors [1].

PCAGC should be distinguished from apocrine carcinoma from breast or ectopic breast tissue, such as gynecomastia [5]. However, it is difficult to differentiate PCAGC from breast apocrine carcinoma because of some similar immunohistochemical and morphological features [4, 5]. PCAGC is often positive for estrogen and progesterone receptors [2, 3]. In comparison, estrogen and progesterone receptors expression in apocrine breast carcinoma are rare, regardless of grade [2, 5]. Therefore, this immunohistochemical stain pattern can be helpful to differentiate these tumors, and serial sections of the whole resected specimen should be evaluated to prove separation from breast parenchymal tissue. Diagnostic features of PCAGC include the presence of the tumor near the dermis, limitation to the subcutaneous layer, cutaneous apocrine glands near the tumor, and separation from breast parenchyma [3, 4].

The present case report has several issues to discuss. First, the differential diagnosis between cutaneous apocrine carcinoma and breast apocrine carcinoma was difficult. Tumors are usually diagnosed based on histopathological evaluations, and different immunohistochemical stain patterns between the two entities can be helpful for differential diagnosis [2, 5]. However, immunohistochemistry alone is not sufficient to confirm diagnostic criteria [2]. Therefore, integrated assessments of medical history, image study, and macroscopic, microscopic, and immunohistochemistry findings should be performed to accurately evaluate and manage these cases. In this case study, the mass could have been mistaken as breast apocrine carcinoma based on morphology and location because the patient had gynecomastia. However, the tumor was pathologically separated from the breast tissue (gynecomastia). In addition, no other malignancy was found during clinical evaluations. Finally, the clinical findings and test results indicated that the mass was PCAGC, not breast apocrine carcinoma. To the best of our knowledge, this is the first case report to describe ingrowing PCAGC arising from the areola in a male patient with gynecomastia. We identified in situ and invasive components of PCAGC in this patient. Pathological findings showed that most tumor cells were located in the in situ *lesions* and limited to the cystic tumor wall, and there was only a microscopic focus of invasive carcinoma. In cystic PCAGC, meticulous pathologic examination, including multiple sectioning and serial section slide preparations, is necessary to establish the degree of invasiveness.

In addition, if a similar mass with malignant features was identified on a chest CT scan in a female patient, it would have been suspected to be breast cancer, and a breast surgeon would have performed the surgery. However, because the mass developed in a male patient and was considered to be benign on the chest CT scan, the surgery was performed by thoracic surgeons. Because the chest wall tumor includes skin appendage tumors, we recommend that thoracic surgeons understand and be able to manage skin appendage carcinomas such as PCAGC.

The treatment of choice in PCAGC has been known to be surgical excision with clear margins, with or without regional lymph node dissection [1, 2]. However, there are no currently established evaluation or management protocols for PCAGC, and the roles of adjuvant therapy and lymphadenectomy remain controversial [1]. In this case, because there was no invasion or distant metastasis and a clear margin of excision, the patient did not undergo adjuvant therapy. At present, 36 months after the surgery, no recurrence or metastasis has been found.

## Conclusion

We described a primary cutaneous apocrine gland carcinoma in the breast of a male patient with gynecomastia. This case is significant because of the differential diagnosis of PCAGC from breast apocrine carcinoma, which is rarely encountered and so is unfamiliar to thoracic surgeons. In the future, we recommend that thoracic surgeons be aware of the possibility of PCAGC during management of a chest wall tumor, especially near the areola.

## Consent

Written informed consent was obtained from the patient for publication of this case report and any accompanying images. A copy of the written consent is available for review by the Editor-in-Chief of this journal. This case study was approved by the Institutional Review Board of Uijeongbu St. Mary’s Hospital (UC15ZISE0051).
